# The Impact of Thyroid Cancer and Post-Surgical Radioactive Iodine Treatment on the Lives of Thyroid Cancer Survivors: A Qualitative Study

**DOI:** 10.1371/journal.pone.0004191

**Published:** 2009-01-14

**Authors:** Anna M. Sawka, David P. Goldstein, James D. Brierley, Richard W. Tsang, Lorne Rotstein, Shereen Ezzat, Sharon Straus, Susan R. George, Susan Abbey, Gary Rodin, Mary Ann O'Brien, Amiram Gafni, Lehana Thabane, Jeannette Goguen, Asima Naeem, Lilian Magalhaes

**Affiliations:** 1 Division of Endocrinology, Department of Medicine, University of Toronto, Toronto, Ontario, Canada; 2 Division of Endocrinology, Department of Medicine, University Health Network, Toronto, Ontario, Canada; 3 Department of Otolaryngology Head and Neck Surgery, University of Toronto, Toronto, Ontario, Canada; 4 Department of Otolaryngology Head and Neck Surgery, University Health Network, Toronto, Ontario, Canada; 5 Department of Radiation Oncology, University of Toronto, Toronto, Ontario, Canada; 6 Department of Radiation Oncology, University Health Network, Toronto, Ontario, Canada; 7 Department of Surgery, University of Toronto, Toronto, Ontario, Canada; 8 Department of Surgery, University Health Network, Toronto, Ontario, Canada; 9 Division of Endocrinology, Department of Medicine, Mount Sinai Hospital, Toronto, Ontario, Canada; 10 The Freeman Centre for Endocrine Oncology, Toronto, Ontario, Canada; 11 The Ontario Cancer Institute, Toronto, Ontario, Canada; 12 Department of Knowledge Translation, University of Toronto, Toronto, Ontario, Canada; 13 Department of Psychiatry, University of Toronto and University Health Network, Toronto, Ontario, Canada; 14 Department of Psychosocial Oncology and Palliative Care, Princess Margaret Hospital, University Health Network, Toronto, Ontario, Canada; 15 Supportive Cancer Care Research Unit, McMaster University and Juravinski Cancer Centre, Hamilton, Ontario, Canada; 16 Department of Clinical Epidemiology and Biostatistics, McMaster University, Hamilton, Ontario, Canada; 17 Centre for Health Economics and Policy Evaluation, McMaster University, Hamilton, Ontario, Canada; 18 Centre for Evaluation of Medicines, St. Joseph's Healthcare, Hamilton, Ontario, Canada; 19 Division of Endocrinology, Department of Medicine, St. Michael's Hospital, Toronto, Ontario, Canada; 20 School of Occupational Therapy, University of Western Ontario, London, Ontario, Canada; Dalhousie University, Canada

## Abstract

**Background:**

Adjuvant treatment with radioactive iodine (RAI) is often considered in the treatment of well-differentiated thyroid carcinoma (WDTC). We explored the recollections of thyroid cancer survivors on the diagnosis of WDTC, adjuvant radioactive iodine (RAI) treatment, and decision-making related to RAI treatment. Participants provided recommendations for healthcare providers on counseling future patients on adjuvant RAI treatment.

**Methods:**

We conducted three focus group sessions, including WDTC survivors recruited from two Canadian academic hospitals. Participants had a prior history of WDTC that was completely resected at primary surgery and had been offered adjuvant RAI treatment. Open-ended questions were used to generate discussion in the groups. Saturation of major themes was achieved among the groups.

**Findings:**

There were 16 participants in the study, twelve of whom were women (75%). All but one participant had received RAI treatment (94%). Participants reported that a thyroid cancer diagnosis was life-changing, resulting in feelings of fear and uncertainty. Some participants felt dismissed as not having a serious disease. Some participants reported receiving conflicting messages from healthcare providers on the appropriateness of adjuvant RAI treatment or insufficient information. If RAI-related side effects occurred, their presence was not legitimized by some healthcare providers.

**Conclusions:**

The diagnosis and treatment of thyroid cancer significantly impacts the lives of survivors. Fear and uncertainty related to a cancer diagnosis, feelings of the diagnosis being dismissed as not serious, conflicting messages about adjuvant RAI treatment, and treatment-related side effects, have been raised as important concerns by thyroid cancer survivors.

## Introduction

The incidence of thyroid cancer is rising in Canada [1] and the United States [2]–[4]. In the United States, the incidence of thyroid cancer has increased from 3.6 per 100,000 persons in 1973 to 8.7 per 1000,000 in 2002, representing a 2.4-fold increase [3]. Also, in the United Kingdom, age-standardised incidence rates for thyroid cancer have nearly doubled from 1.4 to 2.6 per 100,000 persons between 1975 and 2005 [5]. The case fatality rate of thyroid cancer is low, as the annual number of deaths due to thyroid cancer is approximately 5% of the annual number of newly diagnosed cases in the United States [2]. Recent major and sustained improvements in thyroid cancer-related survival have been reported in Europe over the period of 1991 to 2002 [6]. The most frequent histologic subtype of thyroid carcinoma is papillary, accounting for approximately 80 percent of cases, followed by follicular carcinoma (collectively referred to as well-differentiated thyroid carcinoma, WDTC) [7], [8]. In early stage papillary thyroid carcinoma, Hay et al. from Mayo Clinic Rochester have reported a 20-year case fatality rate of less than 1% [9]–[11]. As the incidence of thyroid carcinoma is rising and the disease-related mortality rate is low, the long-term quality of life of survivors is an important consideration.

Currently recommended treatments of WDTC include thyroidectomy and thyroid hormone, sometimes followed by post-surgical radioactive iodine (RAI) remnant ablation (depending on patient and disease features) [12]–[18]. Various bodies have provided clinical practice guidelines on management of thyroid cancer, with recommendations for adjuvant radioactive iodine treatment in early stage papillary cancer ranging from no radioactive iodine for very early stage disease (such as unifocal tumors <1 cm in diameter) to dose activities of 29.9 mCi to100 mCi [12]–[18]. In a recent prospective registry study of well-differentiated thyroid cancer, the National Thyroid Cancer Treatment Co-operative Group reported that radioactive iodine treatment had been administered in 62% to 75% of individuals in their study cohorts [19].

There are no long-term randomized controlled trials examining the efficacy of RAI remnant ablation in thyroid cancer and there are conflicting reports in observational studies on the benefits of adjuvant radioactive in reducing the risk of thyroid cancer-related mortality and recurrence in early stage disease [20]–[22]. Using propensity analysis in prospective registry data, the National Thyroid Cancer Treatment Co-operative Group did not observe a significant treatment benefit of adjuvant radioactive iodine treatment in patients classified as having post-surgical Stage 1 disease (using a study-specific classification system), for the outcomes of overall survival, disease-specific survival, and disease-free survival, respectively [19]. However, this registry study is limited by relatively short mean follow-up period of 3 years [19]. In a recent physician survey performed in Canada and the United States, physicians' recommendations on the use of adjuvant RAI treatment in early stage papillary thyroid cancer were highly variable, with some of the variability explained by region of practice, physician subspecialty, and academic versus non-academic affiliation [23]. In this survey, strong physician support for the use of adjuvant radioactive treatment in early stage thyroid carcinoma was related to beliefs that this intervention: 1) decreases disease-related mortality and recurrence, and 2) facilitates disease follow-up at low risk of adverse effects [24].

In several recent studies, thyroid cancer survivors have been reported to have impairments in long-term health-related quality of life, in spite of cure of disease [26]–[28]. The reasons for impaired quality of life in thyroid cancer survivors in whom the cancer has been cured are not well-understood are not related to thyroid stimulating hormone (TSH) levels [27]. It is not known if a history of RAI treatment has any impact on future long-term quality of life of WDTC survivors nor how the current controversy about the utility of adjuvant radioactive iodine in early stage WDTC impacts patients' perceptions about RAI treatment decision-making.

We conducted a qualitative study exploring patients' perceptions of the WDTC, with particular emphasis on the events related to adjuvant RAI treatment. In general, we asked thyroid cancer survivors what information about their experience would be important to share with future patients and healthcare providers. We explored their recollections of counseling encounters with healthcare providers related to decision-making regarding adjuvant RAI treatment. WDTC survivors provided us with recommendations on how RAI treatment-related counseling may be improved for future patients.

## Methods

### Objectives

Our objective was to explore, from the perspective of thyroid cancer survivors, the experience of a diagnosis of thyroid carcinoma, counseling and decision-making related to adjuvant radioactive iodine treatment, and any short- or long-term consequences of such treatment. We hypothesized that some thyroid cancer survivors would have received conflicting messages about the utility of RAI treatment in early stage WDTC and that a minority of individuals would have suffered side effects from the treatment.

### Participants

All participants were recruited from University Health Network and Mount Sinai Hospital in Toronto, Canada through poster advertisements in Endocrinology, Otolaryngology, Head and Neck Surgery, and Endocrine Oncology clinics. Participants were eligible if they had well-differentiated papillary or follicular thyroid carcinoma (or variant) that was completely resected at primary surgery and if they were offered adjuvant RAI (regardless of whether or not RAI was ultimately accepted and where the treatment was performed). The definition of completely resected thyroid cancer was the lack of known visible residual cancer identified at the time of surgery.

### Description of procedures or investigations undertaken

An in-depth qualitative study was performed, using focus groups to allow participants to build upon ideas raised by other participants [29]–[32]. We conducted three focus group sessions in July, 2007. Each group included five or six WDTC survivors, for a total of 16 participants. All sessions were moderated by a medical facilitator and a qualitative researcher. Participants were instructed that the main topic of interest for the group was the experience of counseling, decision-making, and treatment related to radioactive iodine but that participants could discuss any component of the disease trajectory if they wished. Each session began with a general, in-depth discussion on the personal experience of thyroid cancer, followed by five questions (Appendix S1). The questions for discussion were generated by a panel of content experts including endocrinologists (AMS, SRG, JG SE), radiation oncologists (JDB, RWT), head and neck oncology surgeons (DPG, LR), and three thyroid cancer survivors (Appendix S1). The questions were reviewed by experts in qualitative research (MAO, LM, SS), a psychiatrist (SA), and experts in health research (AG, LT). The questions were presented to participants in the focus group sessions to generate discussions of personal experiences of survivors. The proceedings were audio recorded and transcribed per verbatim (by AN). Additional notes were taken during the sessions by two observers (AMS and AN).

The practice of coding transcribed data involved initially exploring for responses related to the general research questions and then coding the data for respondents' meanings, feelings, and actions [33]. The content of the transcripts was systematically coded using N Vivo 7.0 software (by LM). The data were examined for processes and relationships between specific events and general processes [33]. Coding data led to new categories and more data were collected on the developing categories, upon reviewing all transcripts [33]. Newly gathered data from each focus group was continually compared with previously collected data from prior sessions and their coding [33].

The data analysis was based in grounded theory [34], [35], which relies on the process of constant comparison of qualitative data (statements) retrieved from participants. The constant comparative method is comprised of four stages: 1) comparing incidents (participant statements) applicable to each theme that emerges from the data, 2) integrating themes and their properties, 3) delimiting the theory arising from the themes, 4) and reporting the theory [36]. Theoretical saturation of themes is achieved when no new insights are obtained, no new themes identified, and no issues arise regarding a category of data, in spite of replicating the study conditions (eg. analyzing data from additional focus group sessions) [36]. In this study, the comparative analyses were performed by LM; the identification of themes and theories was performed by LM. The identified themes from the qualitative analysis were then reviewed with an observer with content expertise who was present at all sessions (AMS). The clinical context of themes was clarified by discussion between LM and AMS and any queries were clarified by consulting the original transcripts of sessions. There was final consensus on the identified themes by LM and AMS. The identified themes were also verified by another observer (AN) who was present at all sessions. Complete agreement was achieved on identified themes by the qualitative researcher conducting the sessions and analyzing the data (LM) and the two observers (AMS, and AN). Theoretical saturation of themes was achieved in this study upon analysis of the data from the three sessions. No a priori sample size was required for this qualitative study [37], as adequacy of sample size was defined by saturation of identified themes [36], [37].

Descriptive information on patient characteristics was self-reported by initial telephone interview and self-administered, written questionnaire. The number of individuals reporting one or more RAI treatment-related side effects in the focus group sessions was quantified, using data from the original transcripts. The side effects were quantified by AN, reviewed by AMS, and any clinical queries were resolved by reviewing the original transcripts. Agreement was achieved on the specific side effects identified during the sessions by AMS and AN. We did not quantify other conceptual themes identified in the focus group discussions. Clinical and pathological data was verified in the University Health Network and Mount Sinai Hospital medical records, if allowed by the participant and if the data was available.

### Ethics

Written, informed consent was obtained for participation in the study from all participants and the study was approved by the University Health Network and Mount Sinai Hospital Ethics Review Boards. In an effort to ensure that participants felt comfortable to freely discussing aspects of their medical care in a safe research environment, participants were provided the option of declining consent for the investigators to review of their individual medical records or to have contact with their treating physicians.

## Results

### Description of the Participants

There were 16 participants in the study, twelve of whom (75%) were women. The mean age of participants was 44 years (range 28 to 75 years). Approximately three-quarters of participants had a university education or higher (12/16). The mean time since the diagnosis of thyroid carcinoma was five years (range 2 to 8 years). None of the participants were known to have a history of their thyroid cancer presenting with distant metastases. For the 10 individuals for whom pathologic data was available, primary tumor sizes ranged from 1 to 4 cm, regional nodal metastases were known at time of initial surgery in two individuals, and three individuals had some degree of extra-thyroidal invasion of the primary tumor. The majority of participants (94%, 15/16) had received RAI treatment and none received external beam radiation therapy. Thyroid hormone was withdrawn prior to all RAI treatments. For the 10 individuals for whom data was available on the initial RAI treatment dose, the activities ranged from 100 to 106 mCi. Six individuals chose not to allow contact of their treating physicians nor a review of their medical records to avoid any potential risk of disclosure of their participation to them or their institutions. Approximately one third of the participants in the study had experienced recurrence of disease – five individuals with local-regional recurrence and one who developed new lung metastases. All individuals in whom WDTC recurred received a second therapeutic dose of radioactive iodine.

### Conceptual Themes Identified in the Focus Groups

#### The experience of being diagnosed with thyroid cancer

There were multiple conceptual themes identified in the focus group sessions (Table 1). In each session, participants began with a discussion of what the diagnosis of thyroid cancer meant to them. The experience of being diagnosed with thyroid cancer was felt by everyone to be life-changing and was accompanied by feelings of fear and uncertainty about the future. Participants were generally aware that thyroid cancer-related mortality rates are relatively low and received the message from healthcare providers or the media that they had a “good cancer.” However, the “good cancer” message was generally not considered reassuring to survivors and was perceived as being dismissive of the importance of the diagnosis. The need for life-long follow-up and the possibility of future recurrence of disease was troubling to survivors at the time of diagnosis. Sometimes, participants reported feeling guilt and responsibility for their diagnosis. Participants stressed the need for support from family, friends, and their healthcare team, throughout the experience of being diagnosed with thyroid cancer and undergoing the associated treatments.

**Table 1:** Themes identified in the focus group sessions with thyroid cancer survivors.

Themes	Details
**The life-changing experience of a thyroid cancer diagnosis**	1. The experience of being diagnosed with thyroid cancer changed the lives and the outlook on life of survivors.
	2. The diagnosis was followed by feelings of fear and uncertainty about the future.
	3. Being told that thyroid cancer was a “good cancer” was generally not reassuring to survivors, and was accompanied by feelings that their diagnosis being dismissed as unimportant.
	4. Support from family, friends, and healthcare providers was appreciated.
**The experience of receiving counseling and decision-making on adjuvant radioactive iodine (RAI) treatment**	1. The primary information source related to thyroid cancer treatment, including RAI, was thyroid cancer specialty physicians.
	2. Contradictory messages about the utility of adjuvant RAI treatment were received from physician and internet sources.
	3. Plain-language information about the risks, benefits, and uncertainty about RAI treatment was desired.
	4. The desire for numerical data on disease prognosis and treatment benefits was variable.
	5. Information available on the internet was not considered sufficiently individually relevant.
	6. Individuals varied in their desire to be involved in decision making on RAI treatment.
**The experience after RAI treatment**	1. More than half of participants (8/15) reported some short- or long-term emotional of physical negative effects attributed to RAI treatment.
	2. Side-effects due to RAI treatment were not always recognized by treating physicians at follow-up.

#### Counselling and decision-making related to adjuvant RAI treatment

Participants generally reported that their primary source of information and counselling on adjuvant RAI treatment was their subspecialty physicians, such as endocrinologists, thyroid surgeons, or radiation oncologists. Contradictory messages about the utility of RAI treatment were received from their various subspecialty healthcare providers as well as internet sources. Participants who received information about current clinical practice guideline recommendations (as they applied to their individual case) from their thyroid cancer specialist, greatly valued this communication. Participants generally considered their primary care physicians less knowledgeable and less comfortable counselling and managing thyroid cancer-related medical issues than their subspecialty physicians. The internet was considered easily accessible but the information available on it was generally not considered relevant to their own disease and life situation.

Participants generally wished they had received more plain-language information about the potential risks (short- and long-term side effects), benefits, and uncertainties related to RAI treatment at the time of decision making. For example, concerns were expressed about any potential reproductive implications and second primary cancers related to treatment. The desire for detailed, quantitative statistics on disease prognosis and treatment benefits was variable among participants, with some participants preferring quantitative data and others preferring general descriptive information specifically without numbers. The disclosure of the possibility of thyroid cancer recurrence, in spite of RAI treatment was highlighted by participants as being important. Individuals who suffered a recurrence of thyroid cancer after initial RAI treatment had difficulty rationalizing this outcome and sometimes assigned self-blame. In general, the development and dissemination of, written, plain-language information on risks, benefits, and uncertainty about RAI treatment was endorsed by the groups. The availability of web-based individualized information was also endorsed by individuals who felt comfortable using computers, although additional printed information was still supported.

The desire for personal involvement in RAI decision-making was variable, with some participants preferring to leave the treatment decision to their healthcare providers and others desiring to actively participate in choosing their treatment course. Individuals who preferred an active role in making the decision on RAI treatment, appreciated a sense of control over the experience and feeling like nothing was being imposed on them. In contrast, some individuals felt a sense of disempowerment about RAI decision-making or feeling that there was really no other choice. Some individuals felt most comfortable leaving the ultimate decision on RAI treatment to their treating thyroid cancer physicians, given the medical expertise and experience of their healthcare providers. Only two of sixteen participants indicated that they felt that the decision to accept or decline RAI treatment was primarily their own (after initial consultation with their respective physicians). Family and friends were identified as an important source of emotional support throughout the disease trajectory, although such individuals often lacked sufficient information about thyroid cancer or its treatment.

#### Experiences after RAI treatment

The experience of receiving RAI treatment was reported to be highly variable, ranging from no perceived side effects, to some short- or long-term side effects. More than half of RAI-treated participants (8/15) reported one or more side effects which they attributed RAI treatment, such as: a) short-term effects (nausea, painful salivary gland swelling, changes in taste, sore throat, rash, hair thinning, or menstrual changes) or b) chronic side effects (changes in taste, dry eyes, gum and dental problems). Feelings of isolation at the time of hospitalization for RAI treatment were also reported. The presence of short- and long-term salivary or ocular symptoms after RAI treatment was troublesome to those affected. Primary care physicians and other healthcare professionals sometimes failed to recognize adverse effects of RAI treatment at follow-up, which was frustrating to affected individuals.

Thyroid hormone withdrawal prior to RAI treatment was generally reported to be uncomfortable. Symptoms of thyrotoxicosis due to long-term thyroid hormone suppressive therapy were variable, but when present, palpitations, mood changes, and difficulty concentrating were distressing and sometimes upset relationships and work performance.

#### WDTC survivors' suggestions for the type of information to be shared by healthcare providers in counseling future patients on RAI treatment

In an effort to improve and standardize care for future WDTC patients, the focus group participants provided several key recommendations for healthcare providers to incorporate in counseling patients with WDTC about RAI treatment (Table 2).

- EXPLAIN THE RATIONALE FOR RAI REMNANT ABLATION: Participants felt it was important for physicians to explain the rationale for (or against) adjuvant RAI treatment in their particular case.
- EXPLAIN THE POTENTIAL BENEFITS AND RISKS OF RAI TREATMENT AND RELATED UNCERTAINTY: Participants believed it was important for thyroid cancer specialists to clearly explain, in plain language, the potential benefits as well as the short- and long-term potential adverse effects of RAI treatment and any treatment-related uncertainty (due to a limited high quality evidence) or existing controversy. The provision of written plain language information on risks and benefits of RAI treatment was preferred by many participants. Reproductive implications and second cancer risks were identified as a couple of key areas for discussion.
- TEAM-BASED THYROID CANCER CARE AND AVOIDANCE OF CONFLICTING RECOMMENDATIONS AMONG HEALTHCARE PROVIDERS: A multi-disciplinary team-based, individualized, approach to treatment of WDTC was favoured by participants. Open communication among speciality healthcare providers and individualized treatment recommendations were valued. Some participants expressed a desire to play an active role in the informed decision-making process, whereas others felt comfortable with following their physicians' recommendations.
- INFORMATION SHARING ABOUT CURRENT CLINICAL PRACTICE GUIDELINE RECOMMENDATIONS: Participants greatly valued the expertise of the authors of clinical practice guidelines and indicated that it is important for healthcare providers to explain current guideline recommendations to future patients being offered RAI treatment. A discussion of the application of the clinical practice guidelines to the individual case was valued.

**Table 2:** Recommendations of focus group participants for physician counseling of future patients about adjuvant radioactive iodine (RAI) treatment.

Themes	Details
The rationale for adjuvant RAI treatment	An explanation for the rationale for (or against) adjuvant RAI treatment was desired at the individual case level
The potential benefits and risks of adjuvant RAI treatment and related uncertainty	Plain language information was desired on the benefits and risks of RAI treatment as well ass any uncertainty due to limited high quality evidence or controversy. Potential reproductive implications (for those interested in this) and risk of second primary cancers were emphasized as important areas for discussion. The importance of highlighting that disease may recur in spite of RAI treatment was also suggested.
Team-based thyroid cancer care and avoidance of conflicting recommendations among healthcare providers	A multi-disciplinary team-based, individualized, approach to treatment of WDTC was favoured by most participants. Open communication among speciality healthcare providers and individualized treatment recommendations were valued.
Information sharing about current clinical practice guidelines	A discussion about current clinical practice guidelines as they relate to the individual case was valued by participants.

#### Long-term follow-up and care of WDTC survivors

Participants who reported treatment-related side effects that were not recognized or acknowledged by their healthcare providers reported feelings of frustration. Thus, some individuals sometimes sought second opinions from other thyroid cancer specialists. Thus, an important message from thyroid cancer survivors was the need for legitimization of their treatment-related symptoms at long-term follow-up. Furthermore, participants highlighted the need for long-term support for not only themselves, but also their family, throughout the disease trajectory. Participants stressed the need for more clinical research in the treatment and outcomes in well-differentiated thyroid cancer.

## Discussion

In summary, the participants in our study reported a significant impact of a diagnosis of thyroid carcinoma and its treatments on their lives. The key source of information about WDTC and its treatment that patients identified was their thyroid cancer subspecialty physicians (such as endocrinologists, surgeons, or radiation oncologists). The internet was not deemed an important source of individually relevant information about thyroid cancer, consistent with recent reports that internet information on thyroid cancer is often outdated and incomplete [38]. Fear and uncertainty related to a thyroid cancer diagnosis, a perception of the diagnosis being dismissed as not “serious,” controversies and conflicting messages about adjuvant RAI treatment, and treatment-related side effects, were challenges reported by participants. The amount of detailed information that was desired by participants in counselling encounters, particularly relating to “the numbers” (statistics on prognosis and treatment benefit) was highly variable. Furthermore, the desired degree of active involvement in choosing RAI treatment was highly variable among participants. An approach of evidence-informed team-based, individualized thyroid cancer care was favoured by participants. Based on their unique perspective, participants in our study provided us with some recommendations to improve patient counselling on adjuvant RAI treatment for WDTC. Also, participants highlighted the importance of recognition and validation of treatment-related side effects by healthcare providers at long-term follow-up. The importance of support for family members was also ascertained. A need for more clinical research in the treatment of WDTC was also affirmed.

The strength of this study is its qualitative design with an in-depth examination of the experience of thyroid cancer and its treatment, from the perspective of survivors. Our reasons for choosing focus group methodology for this study are similar to those expressed by Allen et al. in studying breast cancer survivors who received adjuvant treatment [39]. These reasons include: a) inclusion of multiple participants to allow a range of perspectives to be expressed about a phenomenon, b) allowing each participant to contemplate the extent to which he/her experience is similar or different to other group members, and c) allowing participants leeway to express perceptions and feelings that may lead to discussion themes and ultimately illuminate the key aspects of the phenomenon of interest, and d) the collective context of focus groups enabling identification of pertinent themes. The identification of pertinent themes may take longer to emerge through individual interviews (relative to focus groups) and may not be revealed in a structured questionnaire [39].

Many of the themes extracted from our discussions with thyroid cancer are similar to themes reported in qualitative studies of other patient groups. For example, Allen et al. reported that women with a history of early stage breast cancer who received adjuvant treatment expressed fears about disease recurrence and uncertainty about the future [39]; the same sentiments were expressed by thyroid cancer survivors in our study. It is interesting to note that conflicting messages about treatment were not reported in the study by Allen et al. in the field of early stage breast cancer, likely reflecting the wealth of high quality randomized controlled trial data available in that field. In contrast, in the field of early stage thyroid cancer, conflicting findings of observational studies on adjuvant RAI treatment [22], may contribute to the mixed messages about the utility of treatment.

The themes relating to an individual's role in cancer treatment decision making in this study were similar to those previously reported for other disease conditions. In our study, physicians' opinions about their medical condition and potential treatments were highly valued by participants. However, most participants reported that they were not involved in decision making on adjuvant RAI treatment. Of note, patient preference for involvement in treatment decision making was highly variable, with some individuals strongly preferring an active role and others preferring to leave the decision to their physician. In a qualitative study examining women's perceptions about treatment decision- making for ovarian cancer, Elit et al. [40] also reported that most participants did not feel actively involved in a shared treatment decision-making process. In terms of treatment decision making, in a qualitative study of women aged 65 years and older who were diagnosed with breast cancer, Kreling et al. also reported that many women felt they had no choice and followed physicians' treatment recommendations about treatment [41]. In a focus group study of cardiac patients aged 56 years and older, participants indicated that they preferred to follow the cardiologist's recommendation for treatment, based on their medical expertise [42], similar to a view on treatment decision making expressed by some of our participants. Similarly, in a recent qualitative and quantitative study of disease-free rectal cancer survivors and oncologists, Pieterse et al. reported that the majority of patients and clinicians thought that not all patients are able to participate in treatment decision making [43]. In this study of rectal cancer survivors, both patients and clinicians also thought that the clinicians are not always able to weigh the pros and cons of treatment for patients [43]. Pieterse et al. concluded that clinicians should extensively inform patients about their treatment options [43]. The patients in our study also recommended that physicians should explain the potential benefits and risks of treatment even though many of them wanted their physician to provide a treatment recommendation. In reviewing the results of our study and the literature, it appears that individual preferences on involvement treatment decision making are highly variable, although in general, information about the pros and cons of treatment are strongly desired by individual patients.

Some short- and long-term side effects of RAI treatment were acknowledged by participants in this study. Examples of long-term side effects attributed to RAI treatment included changes in taste, dry eyes, gum or dental problems. The issue of long-term and late side effects experienced by cancer patients is receiving increased recognition in the cancer care literature. The Institute of Medicine published an extensive report, “*From Cancer Patient to Cancer Survivor: Lost in Transition*” [44]. One of the key recommendations in this report was that “patients completing primary treatment should be provided with a comprehensive care summary and follow-up plan that is clearly and effectively explained” [44]. One component of such a survivorship plan is a treatment summary that includes an explanation of expected recovery of acute treatment side effects [44]. Another component of a survivorship plan is an ongoing care plan so that cancer survivors receive coordinated care from team members knowledgeable about the disease process and treatment-related side effects [44]. In applying these recommendations from the Institute of Medicine to the care of thyroid cancer survivors, it is hoped that the uncertainties and potential anxiety related to the disease course and any treatment-related side effects may be reduced.

### Limitations

There are several limitations to our study. Firstly, the generalizability of our findings maybe limited because the participants were recruited from only two tertiary/quaternary care academic institutions in the same city. The demographic characteristics of our participants (approximately three-quarters women and mean age in the mid-forties), are in keeping with general population thyroid cancer statistics (76% of new thyroid cancer cases are in women in Canada [1] and the mean age of diagnosis in the Surveillance, Epidemiology, and End Results Program in the United States is 46 years [4]). All participants in this study were English-speaking and many of them were highly educated, which may limit the applicability of our findings to other populations. Moreover, the prevalence of disease recurrence in our study group (approximately one-third), was higher than one would expect expected for early stage papillary thyroid cancer (an average cumulative incidence of 9.3% at 10 years) [22]. In our study, more than half of participants reported one or more short- or long-term side effects attributed to RAI treatment, which is higher than the 29% rate of short-term side effects reported in the thyroid hormone withdrawal group for a recent efficacy study of remnant ablation [45]. It is possible that there may be some recruitment bias in our sample as individuals with recurrent or more complex disease features may have been more likely to be followed in the tertiary/quaternary care environment of our institutions than in the community. Also, some individuals who were initially treated at other institutions may have been referred to our institution for treatment of recurrent disease. Some of the treatment-related side effects reported by participants may have been a reflection of more intensive therapy administered in a tertiary care environment where complex cases are often seen. Also, it is possible that participants who had particularly negative experiences after their diagnosis of thyroid carcinoma or its treatment chose to participate in this study, potentially resulting in some biased findings. Furthermore, it is important to note that all participants in our study who were treated with radioactive iodine underwent thyroid hormone withdrawal, so the RAI treatment experiences may not be generalizable to patients pre-treated with recombinant human thyrotropin prior to radioactive iodine treatment. Our data are also limited by the lack of access to original pathologic data and RAI treatment details for all participants as some participants preferred for us not to access their medical records not contact their treating physicians, in order to ensure a safe environment for discussion of sensitive issues related to their medical care. Another limitation in this study, is that in contrast to a purely quantitative approach, such as a questionnaire, data were extracted from a relatively small number of subjects (albeit sufficient for qualitative analysis), and the results cannot be subjected to a statistical test. Thus, quantitative studies may be useful to determine the prevalence of some of the phenomena identified. For example, with respect to treatment-related side effects, a larger study including patients from multiple institutions may be instructive in better identifying the treatment-related risk relative to dose activity of RAI received.

### Concluding remarks and future research directions

In the current study, we have tried to fulfil the general aim of qualitative research to enhance the awareness of social dynamics in the clinical setting [46], in this case specifically related to the care of individuals with thyroid cancer. We have also highlighted the need for improvement in knowledge translation about adjuvant radioactive iodine to individuals being offered this treatment. In terms of future research and care directions, the desire for more information and stronger evidence about treatment benefits and adverse effects emerged from the focus groups that we conducted. A long-term randomized controlled trial of adjuvant RAI remnant ablation in early stage WDTC would be of great value to generate information of this kind and better inform clinical practice. Moreover, in the mean time, reasonable disclosure of treatment risks, benefits, and uncertainties of adjuvant RAI to future patients should be emphasized, with sensitivity to the individual's specific information needs and avoidance of conflicting messages among healthcare providers. Healthcare providers treating thyroid carcinoma should be aware of the existing clinical practice guidelines relating to the use of adjuvant RAI treatment, and disclose the current recommendations, as they relate to the individual patient's case. Davis et. al have previously reported that reasons for variable implementation of clinical practice guideline recommendations may include the following: the qualities of the guidelines, characteristics of the health professional, characteristics of the practice setting, incentives, regulation, and patient factors [47]. Furthermore, Davis et al. have found that effective strategies for the implementation of clinical practice guideline recommendations may include: reminder systems (such as posters, laminated cards, or structured disease management reminder sheets), academic detailing of an individual physicians by other physicians, and multifaceted interventions (involving two or more interventions such as mailed materials with follow-up phone calls, presentations at meetings, and follow-up meetings with physicians) [47]. Perhaps in the future in the field of thyroid cancer, it may be feasible for organizations publishing clinical practice guidelines to consider developing abbreviated reminder cards or disease management reminder sheets that may enable clinicians to implement current recommendations. However, at present, conflicting observational evidence and the lack of long-term randomized controlled trials on interventions for the treatment of thyroid cancer, makes evidence interpretation and application of clinical practice recommendations very challenging.

Open communication between healthcare providers and patients is an important priority in medical care. Yet, communication with patients about complex treatment interventions may be challenging for physicians. Decision aids are tools or instruments used to inform patients about available treatment options, including evidence about benefits and risks of interventions [48]. Decision aids facilitate evidence-based patient choice [49]–[51] and are useful in the clinical setting when there is more than one reasonable treatment option [52]. In a recent Cochrane systematic review, decision aids were found to improve general patient knowledge, result in more realistic patient treatment expectations, increase the proportion of people active in decision making, and reduce indecisiveness, when compared to usual care [53]. In oncology, decision aids have been typically used to supplement and complement the informal counseling provided by a healthcare provider [54]. For example, in the adjuvant setting, a patient who has had complete resection of malignancy but is at risk for future recurrence; decision aids may facilitate informed decision making about more than one adjuvant treatment alternative, including declining therapy. Decision aids for adjuvant therapy in breast cancer have been successfully developed and tested [55]–[62]. We are currently in the process of developing a computerized decision aid for counselling individuals with early stage papillary thyroid carcinoma on the options of accepting or declining adjuvant radioactive iodine treatment. The intention of such a decision aid is not to replace the importance of physician counselling, but instead to supplement it and facilitate further discussion between healthcare providers and patients. It is also important to highlight that communication between thyroid cancer survivors and their healthcare provider should be facilitated, not only at points in therapeutic decision making, but throughout the entire disease trajectory. The thyroid cancer care team should enable discussions of any disease or treatment-related concerns and address any ongoing needs for emotional support of survivors. The application of survivorship care plans, as outlined by the Institute of Medicine [44], may be of great value in the field of thyroid cancer. The impact of knowledge translation strategies as outlined herein on the physical, mental, and emotional health of thyroid cancer survivors deserves further study in the future.

## Supporting Information

Appendix S1 (0.03 MB DOC)
