# Supplementary material for: The Impact of Thyroid Cancer and Post-Surgical Radioactive Iodine Treatment on the Lives of Thyroid Cancer Survivors: A Qualitative Study
Source: PLoS One. 2009 Jan 14;4(1):e4191. doi: 10.1371/journal.pone.0004191 (PMC2615133; doi:10.1371/journal.pone.0004191)
Supplement: Appendix S1 — (0.03 MB DOC) [file pone.0004191.s001.doc]

**Appendix S1. Outline of the instructions and questions posed to participants during the focus group sessions**

**Preamble:** At the beginning of the session, the facilitators described the process of the focus group. Participants were reminded that the proceedings would be recorded but that the identities of individual participants would be concealed in the transcripts. Participants were reminded that information obtained from any particular individual will not be shared with his or her physician or anyone outside the study and will be destroyed upon completion of analysis of the study. Participants were told they were free not to answer any question if they did not feel comfortable responding to it. Furthermore, were instructed that if any feelings of psychological or emotional distress arose, they should be communicated to the person leading the focus group or the primary investigator (AMS). Participants were told that if psychological or emotional distress occurred, counseling would be offered from a psychiatrist, psychologist, or counselor (if the participant wished).

**Introduction:** Initially, the primary objective of the study was identified, which was to determine the information needs of thyroid cancer patients considering adjuvant radioactive iodine (RAI) treatment. Adjuvant RAI treatment was defined as the administration of radioactive iodine treatment after thyroidectomy in patients in whom disease has been removed by surgery (i.e. with no known residual grossly visible disease at the time of completion of surgery). Participants were invited to share any general personal experiences about thyroid cancer, however, they were told that several questions focused on RAI would be posed later in the session. Participants discussions on the general topic of thyroid cancer were self-directed.

**Specific questions posed during the sessions:** Following the self-directed general discussion of participants on thyroid cancer, we posed several questions:

- 1. In your opinion, what is the most important information that healthcare providers should share with thyroid cancer patients on the potential risks and benefits of post-surgical radioactive iodine treatment? Please explain.
  2. Why did you take or decline radioiodine treatment after initial thyroid surgery?
  3. Do you think that it is important for patients to have the opportunity to be involved in decision making on the issue of RAI treatment after thyroid surgery? What are the benefits and challenges that you see in having patients being involved in such decisions?
  4. The current scientific evidence on the risks and benefits of post-surgical radioactive iodine treatment is limited by the lack of gold-standard, large-scale, long-term controlled clinical trials, resulting in some uncertainty and clinical controversies. Do you think that it is important for healthcare providers to discuss with patients considering this treatment that there is some uncertainty about it? Why or why not?
  5. Are there any other patient needs or opinions that you think healthcare providers should be aware of when discussing post-surgical radioactive iodine treatment with patients?
